# Neuroelectric Correlates of Pragmatic Emotional Incongruence Processing: Empathy Matters

**DOI:** 10.1371/journal.pone.0129770

**Published:** 2015-06-11

**Authors:** Dorian Dozolme, Eric Brunet-Gouet, Christine Passerieux, Michel-Ange Amorim

**Affiliations:** 1 Laboratoire CIAMS (Complexité, Innovation, Activités Motrices et Sportives), Université Paris-Sud, Orsay, France; 2 HandiResp (Recherches cliniques et en santé publique sur les handicaps psychique, cognitif et moteur), EA4047, Université Versailles Saint-Quentin, Centre Hospitalier de Versailles, Le Chesnay, France; The University of Nottingham, UNITED KINGDOM

## Abstract

The emotions people feel can be simulated internally based on emotional situational contexts. In the present study, we assessed the behavioral and neuroelectric effects of seeing an unexpected emotional facial expression. We investigated the correct answer rate, response times and Event-Related Potential (ERP) effects during an incongruence paradigm between emotional faces and sentential contexts allowing emotional inferences. Most of the 36 healthy participants were recruited from a larger population (1 463 subjects), based on their scores on the Empathy Questionnaire (EQ). Regression analyses were conducted on these ratings using EQ factors as predictors (cognitive empathy, emotional reactivity and social skills). Recognition of pragmatic emotional incongruence was less accurate (*P* < .05) and slower (*P* < .05) than recognition of congruence. The incongruence effect on response times was inversely predicted by social skills. A significant N400 incongruence effect was found at the centro-parietal (*P* < .001) and centro-posterior midline (*P* < .01) electrodes. Cognitive empathy predicted the incongruence effect in the left occipital region, in the N400 time window. Finally, incongruence effects were also found on the LPP wave, in frontal midline and dorso-frontal regions, (*P* < .05), with no modulation by empathy. Processing pragmatic emotional incongruence is more cognitively demanding than congruence (as reflected by both behavioral and ERP data). This processing shows modulation by personality factors at the behavioral (through self-reported social skills) and neuroelectric levels (through self-reported cognitive empathy).

## Introduction

If you know that someone’s biggest dream has just come true, you would expect him or her to be happy, rather than sad or angry. Then, if you see this person showing anger, you would be disturbed by it, because this expression does not match the one you expected. This situation represents what we call pragmatic emotional incongruence, i.e. a discrepancy between a cognitive model of reference and a stimulus, as conceptualized by Forabosco [1]. While such incongruence may be premeditated, for example with a humoristic goal [1], it could also reflect a true anomaly, like a misunderstanding of the emotional context or a psychiatric pathology. Below, we will focus on the case where the cognitive model of reference regards emotional cues and is drawn from a sentential context. Sentential contexts refer to the concept of pragmatics, as do other inferences from language cues [2]. Within the embodiment theoretical framework, which posits that sensorimotor information and introspective states ground cognition [3], reading a sentential context should “activate and combine experiential traces in the mental simulation of the described events” [4] that provide cognitive models of reference. The cognitive models of reference related to the present study can be considered as emotional situation models. They would encode emotional cues [5–8] in addition to the other dimensions (time, space, causation, motivation, focal entity, perspective, background entity and features [9]). Using a verification paradigm, we aimed to assess the behavioral and ERP effects of incongruence between an emotional face and a sentence-based situation model encoding emotional cues. Possible influences of empathic dispositions will also be investigated. Facial expressions were employed because they constitute one of the most relevant stimuli when studying human social interaction [10]. They also are a means of limiting cultural biases, as those from basic emotions have been shown to be universally recognized by human beings [11].

### Incongruence processing in affective text comprehension

Emotional cues that violate a situation model have been shown to slow reading [5], [12] and it has been evidenced that it takes longer to determine that a word is emotionally incongruent than emotionally congruent [6]. Studies assessing affective expectancy violation effects on ERPs often use reading tasks [8], [13]-[15]. These studies focus on the effects of reading an unexpected word in a single sentence [14] or use more complex reading tasks to assess emotional incongruence [8], [13], [15]. Two of these studies [8], [14] reported that affective expectancy violation caused a classical N400 effect and enhanced the amplitude of a late frontal positivity. Another study [13] reported a classical N400 incongruence effect but no incongruence effect on a late frontal positivity. Instead, the authors reported that the amplitudes of the N100 and P200 components were greater for emotional inconsistency than for emotional consistency. According to the authors [13], expectation violation effects on early ERP components (namely, the N100 and P200) could be linked to strong expectancies toward the consistent emotional word. Finally, incongruence effects on ERPs are possibly not systematic, as there is at least one study [15] that did not find any effect of emotional valence incongruence on ERPs.

### Insights of affective face priming incongruence

Facial emotional expressions can be harder to recognize when they are contextually incongruent with the given context than when they are congruent, resulting in more errors [16] and increased response times [10], [16]. However, it remains possible to observe no significant effect of incongruence on either response times or accuracy, while using an affective judgment task about word-priming faces [17]. Although processing pragmatic emotional incongruence may be crucial during a real social interaction, to our knowledge, only one ERP study has presented a sentential context followed by an emotional facial expression rather than a textual target. This study [10] used an affective judgment task in order to assess the integration of sentential emotional contexts and emotional facial expressions of joy and anger. The authors found no context effect on the N400 component. Instead, they reported enhanced amplitudes of the LPP (Late Positive Potential) in the incongruent condition compared to the one in the congruent condition. Similarly, two studies employing affective judgment tasks without the use of a sentence as context reported no classical N400 incongruence effect [16]-[17]. The first one [16] employed emotional faces primed by affective scenes. The authors reported a reversed N400 incongruence effect for happy faces and an enhanced LPP amplitude for incongruent sad faces. The second study [17] used words primed by emotional faces and revealed a reversed N400 incongruence effect.

One study [18] reported a classical N400 incongruence effect, while presenting schematic faces of joy or anger, following (after 500 ms) the words “happiness” or “anger”, in a task similar to a verification task. This result contradicts those of the three studies described above [10], [16], [17].The fact that the paradigm used by Krombholz et al. [18] had more to do with a sentence-picture verification task than an affective judgment task could explain why they reported a classic N400 incongruence effect.

### Self-reported empathy and the incongruence effect

One specificity of situation models referring to emotions is that they might, at least partially, be simulated with the help of cognitive empathy. This is because the cognitive dimension of empathy refers to inferences of someone else’s emotional state that are made by “putting ourselves in their shoes”, which can be considered as perspective taking [19]. We assume that such perspective taking might be done with the help of a simulation process. Social expectations might be modulated by empathy. In this way, van den Brink et al. [20] assessed the influence of self-reported empathy on N400 incongruence effects, using the Empathy Quotient questionnaire (EQ, [19]). They presented (non-emotional) sentences spoken by people whose identity (e.g., adult or child) was either congruent or incongruent with the meaning of the sentences. They reported greater N400 incongruence effects for high rather than low empathizers. In an unpublished study, Rak et al. [21] assessed the effect of empathic dispositions on a task assessing how emotion words are integrated, using the Multifaceted Empathy Test. Words were placed in sentences and were congruent, incongruent or unrelated to the sentence they were in. These researchers studied sentences related to intentional emotion, proprioceptive emotion and physical control, and reported that high cognitive empathizers elicited a stronger N400 incongruence effect for the intentional emotion condition than did low cognitive empathizers. This effect of cognitive empathy was greater in the fronto-central regions. While they showed incongruence effects on a late component (P600), this finding was not affected by empathic dispositions.

To our knowledge, the influence of empathy on cognitive processing of incongruence between a sentential context and a face has not been investigated to date. In order to test how dispositions to cognitive empathy could explain some inter-individual differences in the process of pragmatic emotional incongruence, we used a French translation of the EQ [22], described by Lawrence et al. [23] as having three subscales. According to these authors, the first, labeled “cognitive empathy”, corresponds to “the intellectual/imaginative apprehension of another mental state”. The second subscale, labeled “emotional reactivity”, reflects “the tendency to have an emotional reaction in response to others’ mental states”. The last subscale, labeled “social skills”, refers to social adaptation abilities, which might include several psychological constructs (not clearly defined by Lawrence and her collaborators [23]).

### The present study

We wanted to assess both affective face priming and affective text comprehension, combining them in order to examine their potential relationships with subcomponents of empathy. For this purpose, we used facial expressions of basic emotions, which should help prevent misdetections of pragmatic emotional incongruence caused by misrecognitions of our emotional facial expression stimuli. In order to force participants to cognitively process the context in an emotionally pragmatic way, we created emotional contexts based on sentences that described someone in a situation that would or would not elicit basic emotions of joy, fear, sadness or anger. For example, one of the sentences presenting someone in a joyful context was: “Elle/Il a le sentiment d'avoir complètement réussi sa vie.” (“She/He feels like she/he has been a total success.”). These sentences were presented to the participants, followed by either a face expressing an emotionally neutral face or one expressing an emotion that was either congruent or incongruent with the context. The faces were rendered using a computer 3D-model in order to create customized emotional facial expressions. While it is supposed that two stages are involved in brain processing of incongruence (the detection of incongruence and its resolution [1]), we sought to focus on the detection of incongruence, by asking the participants to judge whether the emotion (or lack thereof) displayed by the face matched the one that could be inferred from the sentence. As the N400 is supposed to reflect pragmatic processing [24], we expected pragmatic emotional incongruence to elicit a classic N400 effect. Such an effect might be enhanced by cognitive empathy [21]. Finally, we expected an incongruence effect on the LPP [10], [16]. Other possible effects of EQ scales are difficult to predict, given the fact that we did not find any study assessing them in a task similar to ours.

## Materials and Methods

### Participants

Thirty-seven participants, fluent in French, took part in the EEG experiment, although statistical analyses were conducted on data from just 32 participants (16 women, 16 men; *M*age: 22.9 years ± 3.5 *SD*). We excluded data from participants having correctly answered less than 70% of each condition or whose data presented too many artifacts, so that there was always at least 50% of retainable trials per condition (i.e. 50 trials). Handedness was assessed using a French version of the Edinburgh Handedness Inventory [25]. Twenty-nine participants were right handed, two left handed and one was ambidextrous. None reported any neurological or psychiatric disease. They had normal or corrected-to-normal vision.

All participants gave informed written consent before the experiment, in accordance with the ethical standards of the Declaration of Helsinki. The EA 4532 local Ethics Committee of Université Paris-Sud approved this study specifically.

### Empathy questionnaire

**Fig 1:**
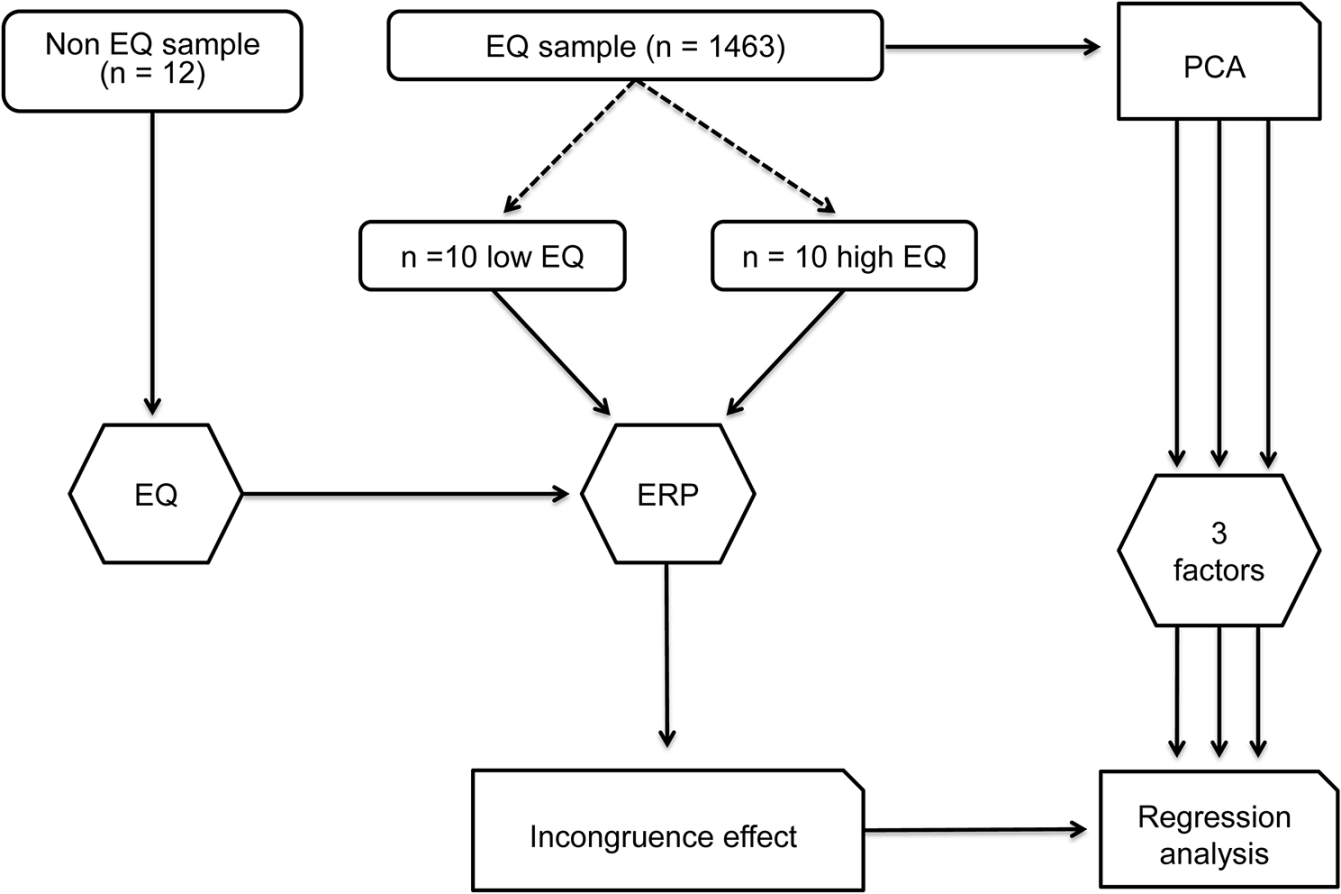
Illustration of the procedure used as regards empathy.

Fig 1 illustrates the procedure for assessing empathy.

Initially, 16 participants performed our task before completing a French translation [22] of the Empathy Quotient questionnaire [19] In order to involve more participants presenting more highly contrasting scores on the EQ, we recruited additional participants based on their scores. To this end, we uploaded the French translation of the EQ questionnaire to internet. There were 835 female and 628 male (*M*age: 23.27 years ±6.13 *SD*) respondents, mostly from Université Paris-Sud. The EQ questionnaire contains 40 empathy and 20 filler items. The scores range from 0 (low empathy) to 80 (high empathy). This questionnaire was cross-culturally validated with healthy subjects and with patients having autistic spectrum disorders [22]. Correlations with neurofunctional data were reported in fMRI [26] as well as both correlations and regressions with EEG data [20].

The 1 463participants completing the online EQ questionnaire obtained an average score of 37.76/80 ± 9.24 *SD* on the EQ. We performed an ANOVA on the Empathy Quotient score, using gender (2) as a factor. On average, women scored significantly higher than men, with 40.06/80 versus 34.67/80 for men, *F*(1,15) = 133.87; *P* < .01, η² = .08.

We screened the respondents in order to recruit the 20 participants with the highest and lowest EQ scores (10 highest, 10 lowest; at least one standard deviation from the average calculated on our 1 463 respondents). The participants who had not previously completed the EQ online completed a printed version of it at the end of the experiment.

The EQ questionnaire contains three subscales [23]: “cognitive empathy”, “emotional reactivity” and “social skills”. The subscale items were chosen according to the factors we found while running a PCA on the 1 463 online respondents to the EQ questionnaire. The results of this PCA are presented in Table 1.

**Table 1:** Results of the PCA made using Lawrence et al. (2004)’s items, with data from 1 463 respondents to the EQ questionnaire.

	EQ Items	Factor 1	Factor 2	Factor 3
Cognitive empathy	19	**0.702**	-0.069	0.058
	25	**0.648**	0.182	0.096
	26	**0.639**	0.143	0.099
	44	**0.597**	0.136	0.070
	52	**0.737**	0.170	0.092
	54	**0.607**	0.046	0.067
	55	**0.738**	0.028	0.053
	58	**0.595**	-0.096	0.005
	41	**0.590**	-0.002	0.086
Emotional reactivity	6	0.150	**0.536**	0.024
	42	0.162	**0.587**	-0.086
	59	0.131	**0.675**	-0.065
	21	0.059	0.442	0.330
	32	-0.051	**0.703**	-0.017
	48	-0.065	**0.644**	0.179
Social skills	4	0.071	0.009	**0.552**
	8	0.135	-0.023	**0.681**
	12	-0.012	0.405	0.448
	14	0.055	0.193	**0.562**
	35	0.129	-0.225	0.418
	Prop. Explained Variance	0.198	0.128	0.083

Loadings of the items used for regression analyses on behavioral and ERP data are in boldface type.

We kept only items with loadings ≥.50 (that is ≥ 25% of explained variance). Items 19, 25, 26, 41, 44, 52, 54, 55 and 58 were considered as assessing “cognitive empathy”. We took items 6, 32, 42, 48 and 59 as representing “emotional reactivity”. Finally, items 4, 8 and 14 were grouped as evaluators of “social skills”. The score for each EQ subscale was computed from the average score on the items representative of the subscale (according to the PCA). On average, the participants in the ERP experiment scored 8.1/18 ± 4.9 *SD* on the cognitive empathy scale, 5.4/10 ± 3.3 *SD* on the emotional reactivity scale and 2.8/6 ± 1.6 *SD* on the social skills scale.

### ERP experiment

During the experiment, the subjects were presented 20 blocks of 10 trials. Each trial consisted of a sentence describing an emotional situation followed by a static emotional face. The subjects were then asked to decide whether the facial emotion was congruent with the situation described in the sentence.

#### Selection of stimuli

All of the stimuli were chosen based on the results of a forced choice validation study (12 participants: 6 women, 6 men, *M*age: 32.7 years ± 14.6 *SD*). The participants were required to categorize emotionally each stimulus as joy, fear, anger, sadness or neutral. An analog scale was also used to test the participants’ perceived emotional intensity (except for neutral stimuli). They were instructed to click on the scale with the computer mouse, from *low emotional intensity* to *high emotional intensity*. There were 10 groups of 25 sentences (250 trials) and 8 groups of 8 faces plus 1 group of 10 faces (74 trials). The groups of sentences and faces were alternated. The sentences were presented for 3 000 ms, the participants were allowed to answer (by clicking on a box) during sentence presentation and had 2 000 additional milliseconds to answer after the sentence disappeared. The procedure was similar for the faces, except that they were displayed for only 1 000 ms.

**Sentences:** The sentences described a situation that would elicit a specific and unambiguous basic emotion in a person such as joy, sadness, fear or anger. Some situations were inspired by the material described in Russ et al. [27]. Neutral sentences (with no inferable emotion) were added. There were 20 different sentences for each emotional category. Each sentence was formulated in the third person with either a male/female or neutral gender, resulting in a total of 200 sentences. Sentence stimuli were 89% (± 8% *SD*) correctly categorized on average. The perceived intensity for emotional (non-neutral) sentences was 68% (± 14% *SD*) on average. The sentences are provided as S1 Sentences

**Fig 2:**
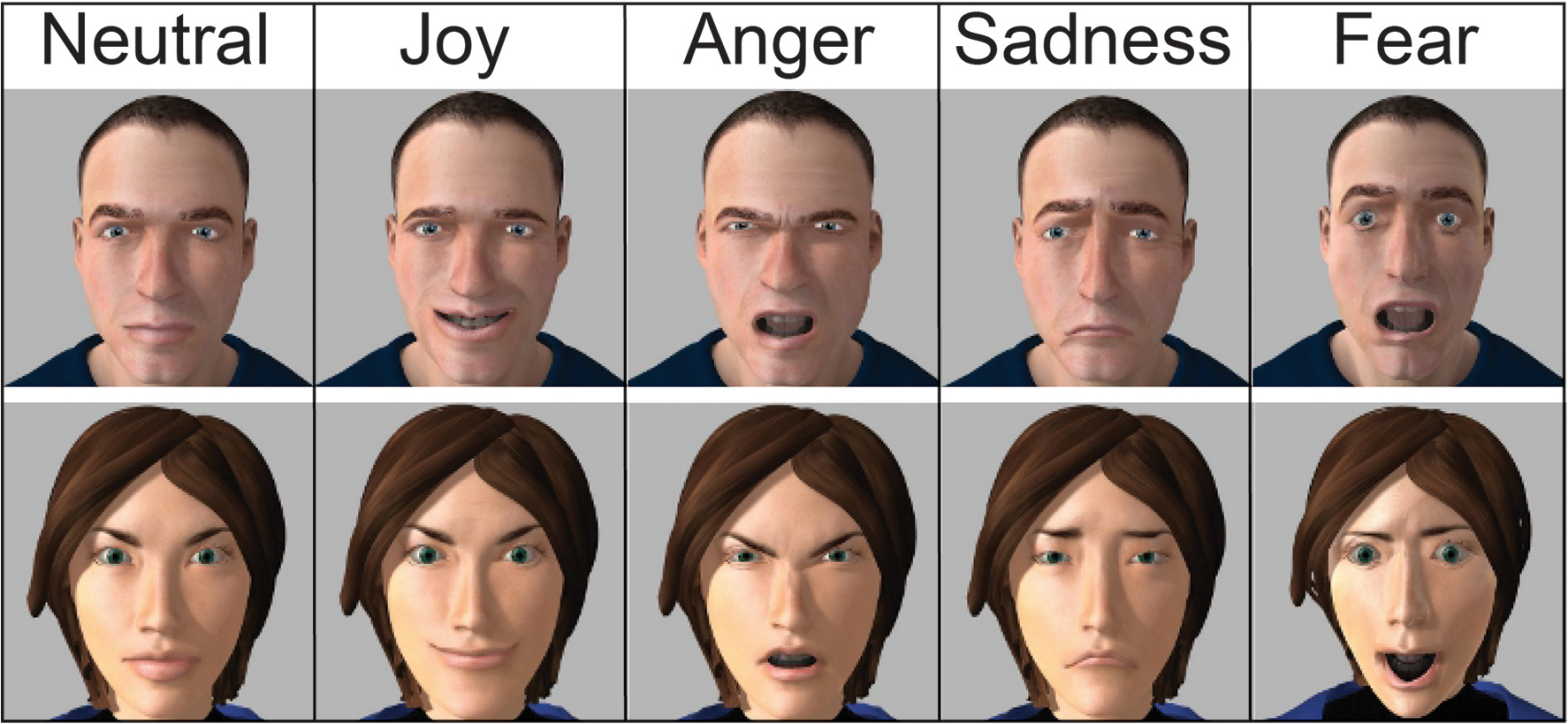
Faces. Facial expressions of joy, anger, sadness, fear and the neutral faces were created using M.A.R.C. software (LIMSI). All of the faces are presented here.

**Faces:** The facial expressions for primary emotions (for a description, see Ekman and Friesen, 2003) of joy, fear, anger and sadness plus neutral expressions were generated on M.A.R.C. software [28]. In order to match the sentences’ gender, one female and one male virtual character was employed. This resulted in ten different facial expressions, one for each emotional category and avatar (cf. Fig 2). The pictures measured 4 cm x 4 cm, corresponding to 4° x 4° visual angles at the participants’ viewing distance. The emotional faces were correctly categorized 83% (± 17% *SD*) of the time on average. The average perceived emotional intensity of non-neutral facial expressions was 69% (± 6% *SD*).

### Experiment

The participants were seated in a dimly lit room in front of a 24-inch LCD monitor, at a viewing distance of 57 cm. They were given oral and written instructions on the experimental task prior to training. The participants were provided ear plugs to prevent noise disturbance, and were asked to keep their gaze directed at the center of the screen. They were instructed to read sentences presenting someone in a particular situation potentially eliciting joy, fear, anger or sadness. They were advised that a situation not eliciting any emotion should be considered as neutral. We informed the participants that a face would be displayed after each sentence, expressing joy, fear, anger or sadness or a neutral expression. We then asked them to judge whether the facial expression matched or mismatched the emotion (or absence of emotion) suggested by the sentence.

The experiment included 20 blocks of 10 trials, randomized among the participants. Fifty percent of the trials were congruent, and 50% were incongruent. The experiment was performed using Eprime 2.0 PST software. The participants started with a set of ten practice trials using stimuli different from the experiment.

**Fig 3:**
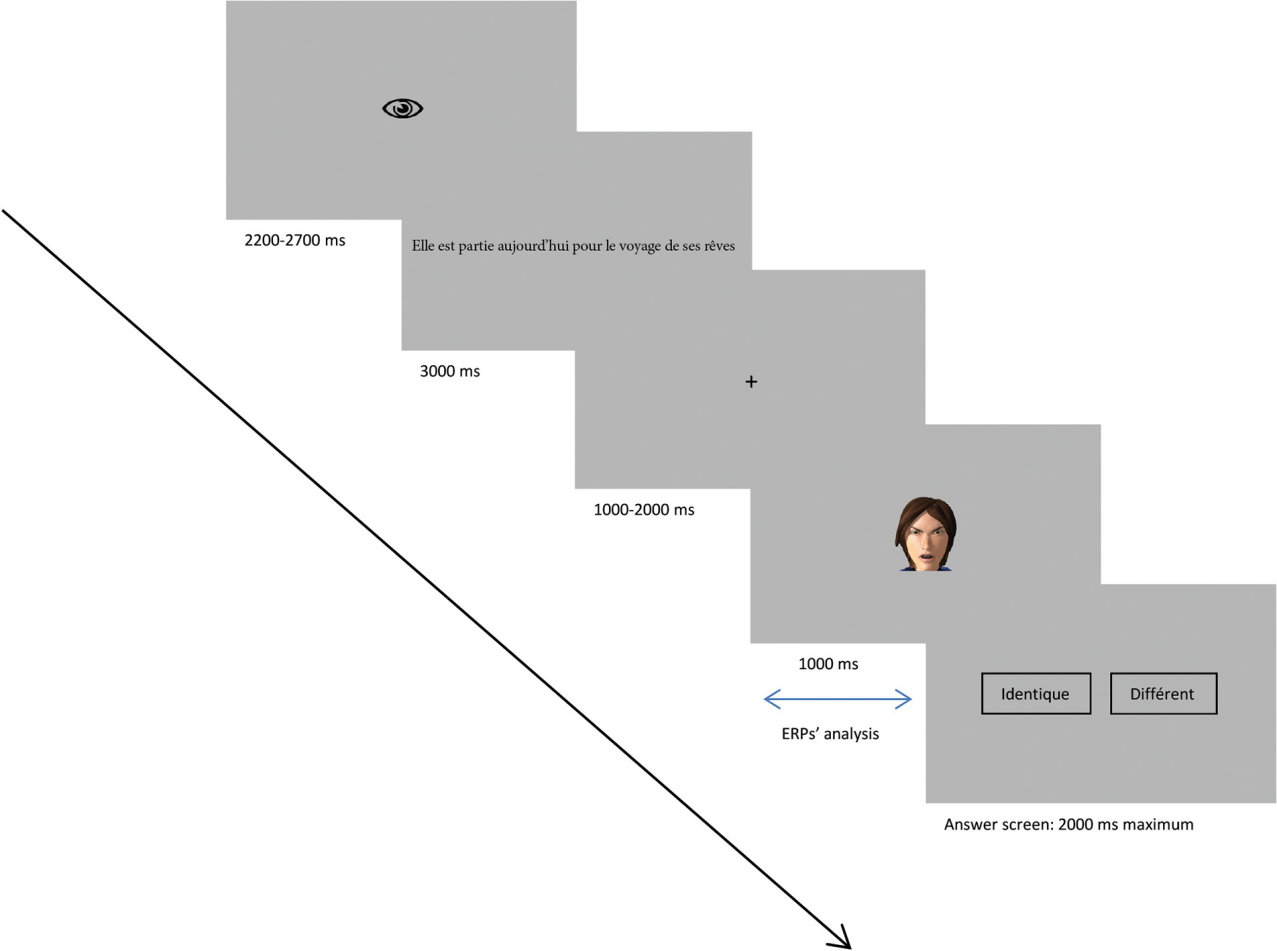
Experimental procedure. A sentence presenting someone in an emotional (joyful, fearful, angry, sad) or neutral context was displayed, followed by a face expressing an emotion (joy, fear, anger, sadness) or no emotion (neutral). Participants were asked to answer “Same” if the emotion expressed by the face corresponded to the emotion suggested by the sentence, and “Different” if this was not the case. Here, the angry face is incongruent with the sentence “Today she left on the trip of her dreams”.

Each trial began with an eye-symbol displayed for a random duration of 2 200 to 2 700 ms inviting the subjects to blink (to prevent blinking during the stimuli display). Then a fixation cross was displayed for a random duration of 1 000 to 2 000 ms. After the fixation cross, a sentence was displayed for 3 000 ms. The fixation cross appeared again, with a random duration of 1 000 to 2 000 ms. Then, a face appeared for 1 000 ms, followed by the answer screen composed of two choices: “identique” or “différent” (resp. “same” or “different”). Each feminine sentence was followed by a female face and each masculine sentence was followed by a male face. The participants were allowed 2 000 ms to answer using number pad keys “1” and “3” (with their right hand). They had to answer “same” if they thought the face was congruent with the sentence and “different” if it was not. The order of the answer keys (and onscreen boxes) was randomized among the participants. The procedure is illustrated in Fig 3.

Each sentence was randomly paired with all facial expressions (by gender) across the participants, without counterbalancing constraint.

### EEG recording

The EEG was recorded by 32 active Ag-AgCl electrodes (ActiCap) placed on the scalp with an elastic cap, and connected to a BrainAmp system (Brain Products). The electrode positions were compliant with the 10–20 system. The sampling rate was 1 000 Hz. No filter was applied during data acquisition. Electrode impedances were kept below 20 kΩ.

Recorded data were then processed by Brainvision Analyzer 2 software, using the FCz electrode as reference (this reference was not used for data analyses). The EEG signal was band-pass filtered (0.1–30 Hz, 12 dB/Octave). Filtered data were segmented into periods from 500 ms before to 1 000 ms after the presentation of the emotional faces. Segmentation was performed within subjects according to experimental conditions (congruent and incongruent). Only segments corresponding to correct answers were analyzed. Automatic artifact rejection was performed to remove segments with a gradient greater than 50 μV/ms, difference (max-min) greater than 200 μV per 200 ms interval and activity of less than 0.5 μV per 100 ms interval. Then a second, semi-automatic artifact rejection was performed to reject segments containing ocular artifact bases upon visual inspection. We could have corrected data for ocular artifacts but chose not to do so as we considered rejecting affected trials as less data disruptive. On average, we rejected 14.6% of trials due to ocular and other artifacts. The number of trials per remaining participant available for statistical analyzes was 79 in the congruent condition and 76 in the incongruent one. A t-test on the number of rejected trials by condition revealed no significant difference (P >.1). Data were re-referenced to the average of all electrodes. Baseline correction was done taking the 200 ms pre-stimulus period as the reference. Finally, the segments were averaged for each participant and condition. We removed 30 ms from all time values for the computer monitor’s 30 ms latency, in order to time-lock ERPs at stimulus onset.

### Data analysis

#### Behavioral data

Repeated measures analyses of variance (ANOVAs) were conducted on behavioral correct answer rates and response times to correct answers, with congruence (two levels) as the main factor.

#### Event-Related Potentials (ERP)

**Fig 4:**
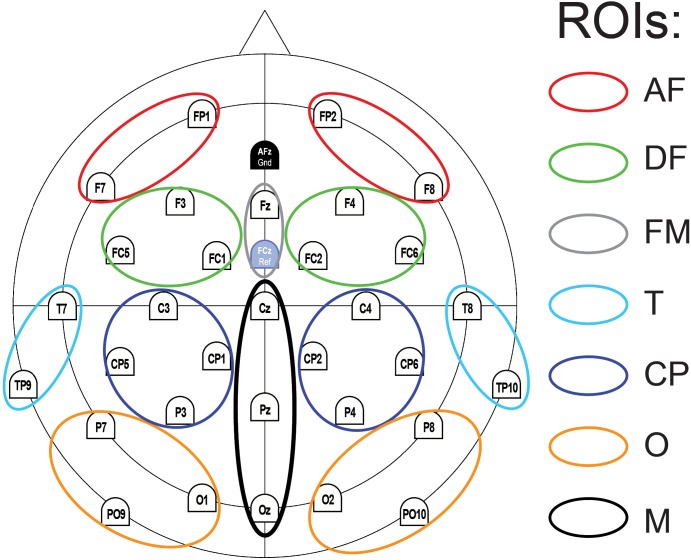
Regions of interest (ROIs). We grouped electrodes in seven ROIs: antero-frontal (AF), dorso-frontal (DF), frontal midline (FM), temporal (T), centro-parietal (CP), occipital (O) and centro-posterior midline (M) groups of electrodes. ROIs were further subdivided by hemispheres (except for M).

First, regions of interest (ROI) were defined according to functional anatomical regions: occipital (O ROI), centro-parietal and parietal (CP ROI), temporal (T ROI), antero-frontal (AF ROI), dorso-frontal (DF ROI), frontal midline (FM ROI) and centro-posterior midline (M ROI). Functionally, the occipital and temporal regions are known to be associated with ERPs reflecting visual processes and related to face processing, concomitant with P100 and N170 waves [29]. The centro-parietal and frontal regions are associated with higher level processes such as context integration, echoed by the N400 wave [24]. Frontal sites were separated in order to keep only electrodes with similarly shaped EEG curves. ROIs are summarized in Fig 4. The following time windows were considered: [50; 120 ms] after face stimuli onsets for the P100 wave; [120; 200 ms] for the N170 wave; [200; 340 ms] for the P300/Early N400 wave; [340; 470 ms] for the N400 wave; and [470; 960 ms] for the LPP.

To assess the incongruence effect, we performed ANOVAs on the amplitude value (μV) for each time window considered, with all ROIs (7 or 12) and congruence (2) as within-subject factors. Note that, for the purpose of these particular ANOVAs, we split each initial ROI (whether O, CP, T, AF or DF) in two: one for the left hemisphere and one for the right hemisphere (except for the M ROI). When a significant “congruence x ROI” interaction was found, we conducted subsequent separate ANOVAs for each concerned time window and ROI. These separate ANOVAs were conducted on the amplitude value (μV), with congruence (2) and hemispheres (2) as within-subject factors. Analysis of Midline ROIs did not involve the hemisphere factor. Planned comparisons were used to disentangle a significant interaction between congruence and hemisphere. These planned comparisons were considered as significant when the *P* values were below .025, according to the Bonferroni correction for multiple comparisons (two comparisons in this case) with an initial α = .05 criterion. When appropriate, Greenhouse-Geisser corrections were applied to the p-values. Because we aimed to study correctly perceived incongruence effects, only correct answers were considered for analyses on ERP data.

#### Analyses of empathy correlates

In order to reveal the different relationships between the empathy subscales and the findings as defined above, we performed regressions on incongruence effects (data value in incongruent condition–data value in congruent condition) with the three EQ subscales as continuous predictors for each wave and ROI, as well as on behavioral data (response time and correct answer rates). We used multiple regression models with forward and backward procedures. We checked for outliers in all significant regressions, using the standard residual method (> 2 * sigma). We observed one outlier for the regression from the O ROI in the N400 time window. Similarly, one outlier was found for the regression from the DF ROI in the N400 time window. Correct answer rates and response time regressions each presented two outliers.

As regards ANOVAs, we considered *P* values below .05 as significant and .05 ≤ *P* < .1 as marginally significant. Regressions were considered as significant when the *P* values were below .0056, according to the Bonferroni correction for multiple comparisons (nine comparisons were done for each analysis) with an initial α = .05 criterion.

## Results

### Behavioral measures

#### Description of the behavioral incongruence effect

Correct answer rates were significantly lower in the incongruent condition (*M* = 88.66%) than in the congruent one (*M* = 93.00%), *F*(1,31) = 6.58, *P* < .05, η² = .18. Likewise, response times were significantly higher for incongruent stimuli (*M* = 631.16 ms) than for congruent stimuli (*M* = 603.84 ms), *F*(1,31) = 4.21, *P* < .05, η² = .12.

**Fig 5:**
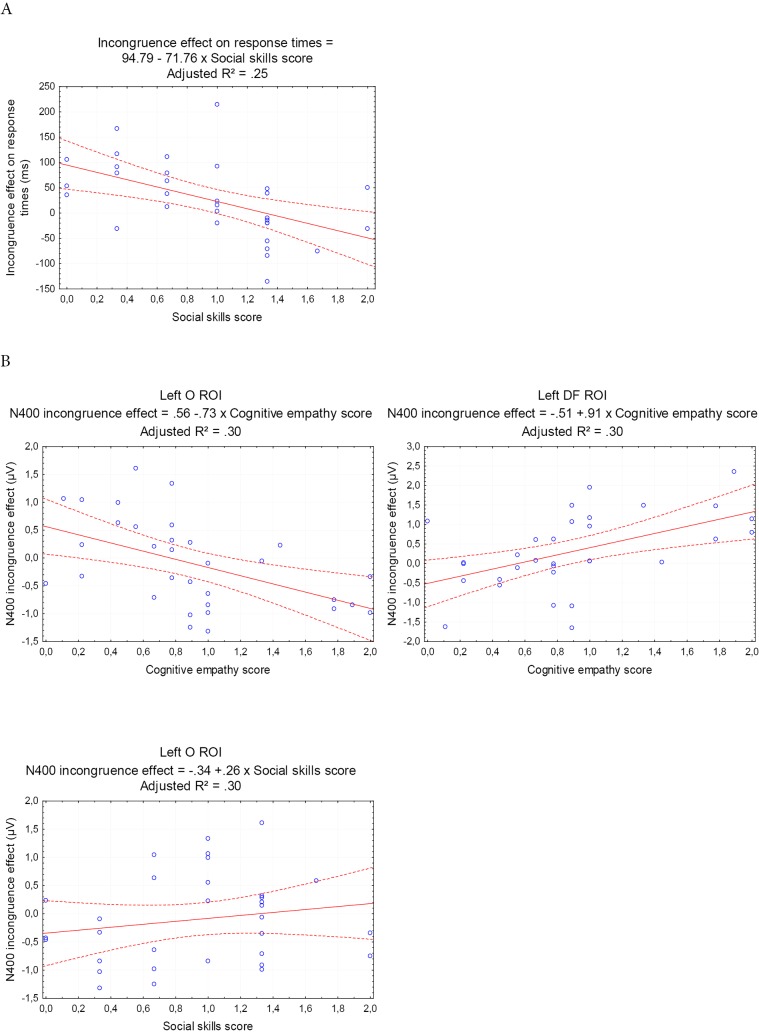
Scatterplots illustrating simple regressions corresponding to significant multiple regressions. (A) Scatterplots of the regression on the response times. Response times tend to be higher as the social skills scores decrease. (B) Scatterplots of the regressions on the N400 incongruence effect, in the left O and DF ROI. In the O ROI, the N400 incongruence effect appears increased for high cognitive empathy scores and linearly decreases until it reverses for low cognitive empathy scores. A reversed pattern is observed in the DF ROI but might be the counterpart of the effect in the O ROI. C: In the left O ROI, the N400 effect also appears to barely decrease as the social skills scores increase.

**Regression of EQ subscales on the behavioral incongruence effect:** The incongruence effect (response to incongruent stimuli minus response to congruent stimuli) on response times was predicted significantly by the scores at the social skills subscale (cf. Table 2): the greater the social skills, the greater the sensitivity to incongruent versus congruent stimuli (cf. Fig 5A).

**Table 2:** Significant regressions on incongruence effects.

Adjusted R² = .20 for RTs				
	Variable	Estimate(SE)	*β*(SE)	Statistics
	Social skills	-78.28(24.47)	-.57(0.18)	*t*(28) = 3.20,*P* = .0034
Adjusted R² = .30 for N400 incongruence effect (μV) in the left O ROI				
Hemisphere	Variable	Estimate	*β*(SE)	Statistics
Left	Cognitive empathy	-0.89(.23)	-.61(.16)	*t*(28) = -3.91,*P* = .00054
Left	Social skills	.72(.22)	.49(.15)	*t*(28) = 3.30,*P* = .0026
Adjusted R² = .30 for N400 incongruence effect (μV) in the left DF ROI				
Hemisphere	Variable	Estimate	*β*(SE)	Statistics
Left	Cognitive empathy	1.24(.31)	.70(.17)	*t*(28) = 4.01,*P* = .00041

### Event related potentials

#### Topography and chronometry of the components

**Fig 6:**
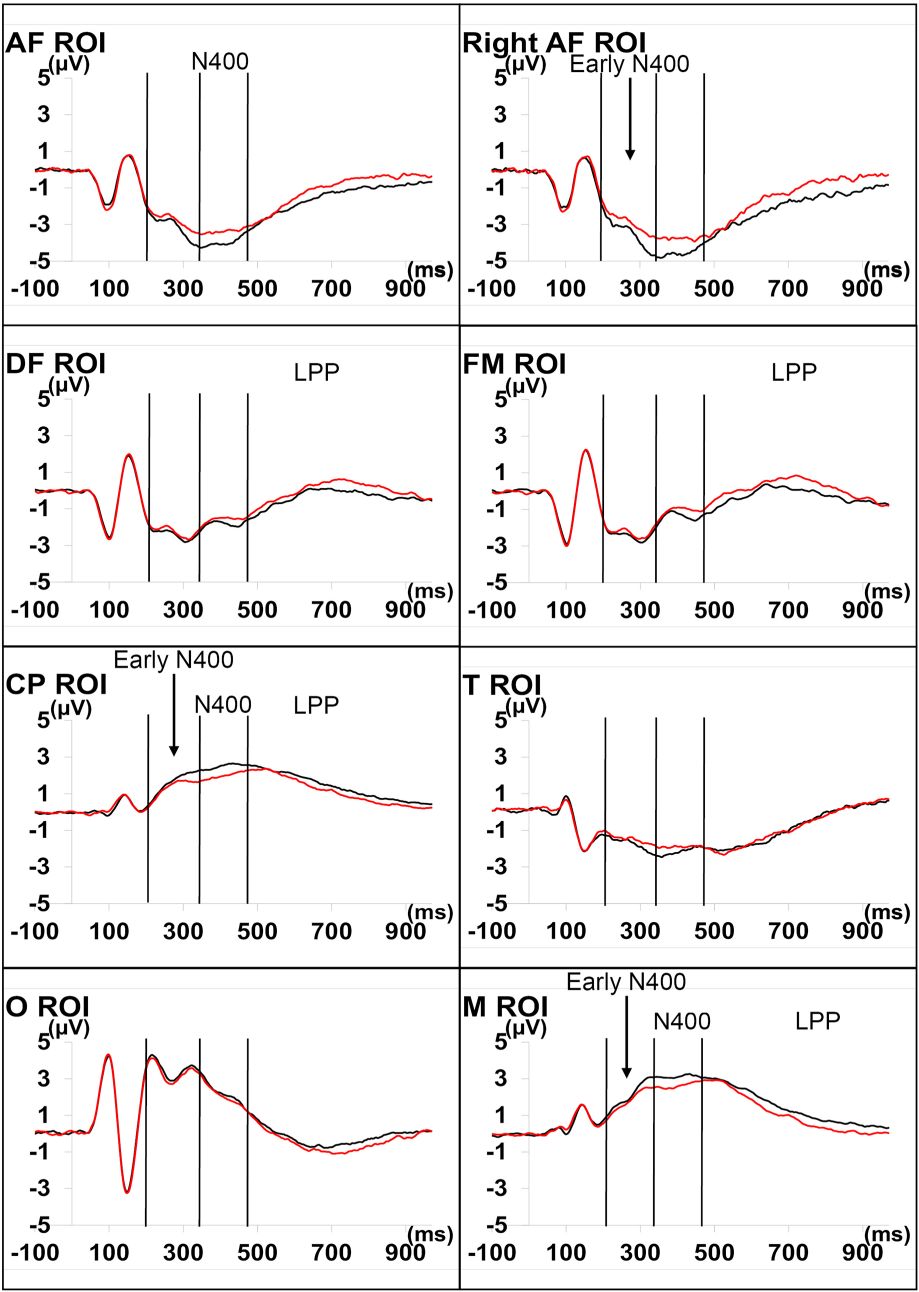
Overall average ERPs for congruent and incongruent conditions (all participants combined). In the CP and M ROIs, the amplitude of the Early N400 is significantly lowered by incongruence. The incongruence-related N400 deflection is significantly lower than the congruence-related deflection in CP and M ROIs. The incongruence-related late wave deflection is more positive-going than the congruence deflection in DF and FM ROIs. Reversed effect observed in the right AF ROI (early N400), AF ROI (N400), CP and M ROIs (LPP) are supposedly electrical counterparts to the effects found in regions where effects are classical.

Independent of the congruence factor, five ERP components were evoked by face onsets (see Fig 6). The first was a P100 wave, positive-going in posterior regions and peaking around 100 ms after stimulus onset in the O ROI. The second was a posteriorly negative-going N170 wave, peaking at around 150 ms in the O ROI. The third was possibly a P300 wave, peaking at around 310 ms in the O ROI. However, apart from the O ROI, it seemed to overlap with the N400. Consequently, we will call it “early N400”. The following component was in the N400 time window. It was not negative-going in the CP and M ROIs but amplitude appeared to be reduced by incongruence in these (CP and M) ROIs. Finally, the last component followed the N400 wave and lasted until around one second after stimulus onset. It appeared as an LPP, especially in anterior brain regions (ROIs AF, DF and FM). Lateralization effects are reported in S1 Supporting Information.

#### Incongruence event-related potentials

**Fig 7:**
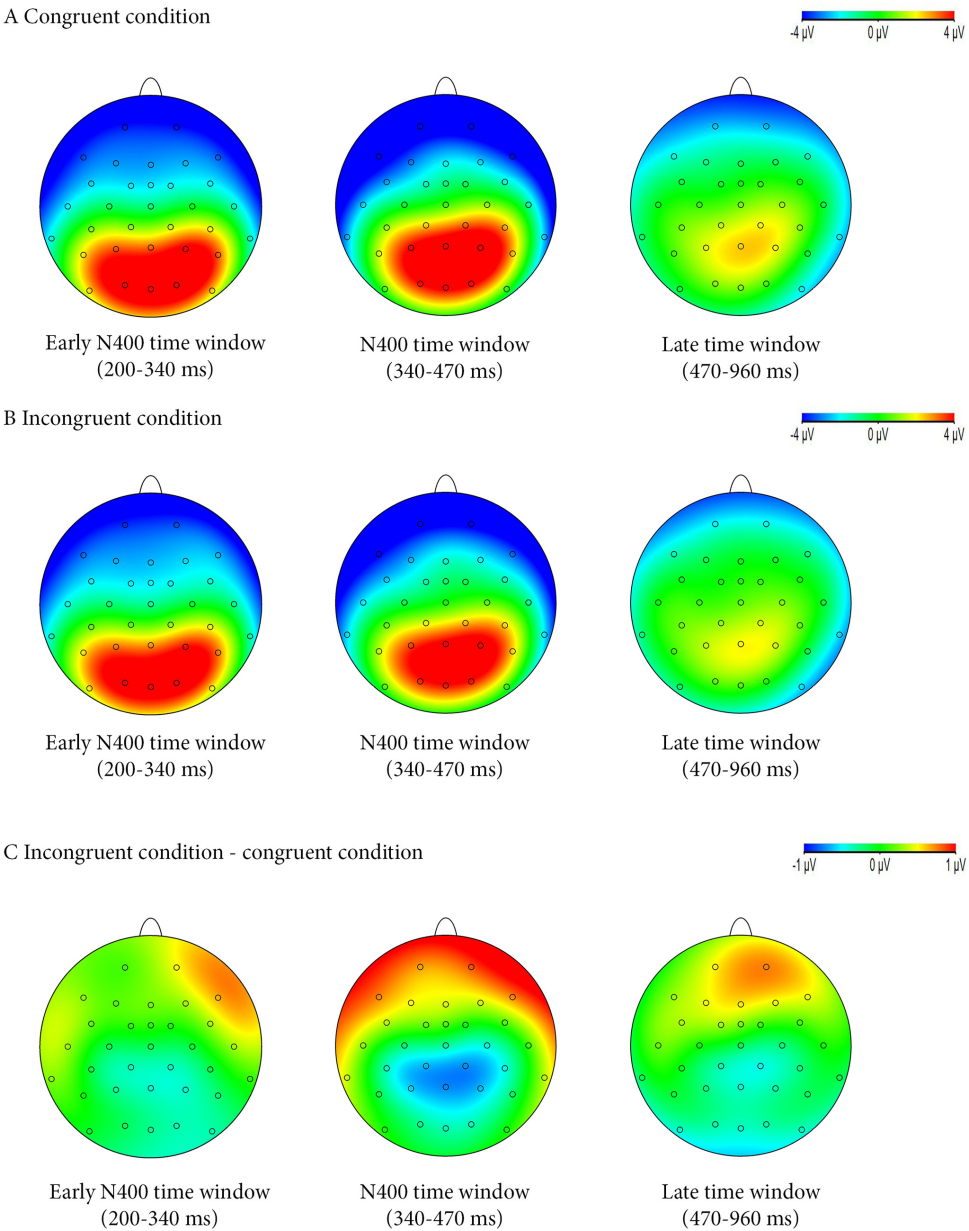
Topographical maps of ERP grand averages. The maps represent the Early N400, N400 and LPP time windows in: A—the congruent condition; B—the incongruent condition; and C—the differences between the incongruent and congruent conditions.

The main effects of incongruence on the ERPs (by ROIs) are reported in Table 3. Potential mean signals are shown in Fig 6, whereas EEG topographies of Early N400, N400 and LPP are presented in Fig 7. There was no incongruence effect on the amplitude of the P100 and N170 waves. Incongruence effects on the ERPs started in the Early N400 time window, where the “congruence x ROI” interaction was significant, *F*(11,341) = 3.57, *P* < .05, ε_g-g_ = .34. In the CP M ROI, the amplitude of the early N400 was significantly lower in the congruent condition than in the congruent condition. A similar effect was found in the M ROI. In the AF ROI, we observed a significant “congruence x hemisphere” interaction, *F*(1,31) = 5.36, *P* < .05, η² = .02. In the right AF ROI (but not in the left one), the amplitude of the early N400 time window was significantly less negative in the incongruent condition (*M* = -2.89 μV) than in the congruent one (*M* = -3.48 μV).As regards the N400 time window, there was a significant “congruence x ROI” interaction, *F*(11,341) = 6.53, *P* < .001, ε_g-g_ = .34. CP and M ROIs exhibited significant main effects of incongruence with more negative-going N400 components in the incongruent condition. The AF ROI presented a significantly smaller N400 component in the incongruent condition than in the congruent condition, while marginal similar incongruence effects in the N400 time window were found in the T, FM and DF ROIs. There was also a “congruence x ROI” interaction in the LPP time window, *F*(11,341) = 3.03, *P* < .05, ε_g-g_ = .29. In the FM and DF ROIs, incongruent faces elicited a significantly more positive-going LPP than congruent faces. This incongruence effect was only marginally significant in the AF ROI. CP and M ROIs presented a significantly reduced amplitude of the LPP in response to incongruent faces. Finally the late incongruence effect in the O ROI was similar to the one observed in the CP and M ROIs but it was only marginally significant.

**Table 3:** Significant or marginal incongruence effects on ERPs.

Wave	ROI	F value	Significance	Amplitude in congruent condition (μV)	Amplitude in incongruent condition (μV)	η²
Early N400	**CP**	*F*(1,31) = 11.85	***P*<.01**	1.54	1.28	.03
	**M**	*F*(1,31) = 7.20	***P*<.05**	2.06	1.77	.20
N400	**AF**	***F*(1,31) = 20.87**	***P*<.001**	**3.99**	**-3.38**	**.10**
	DF	*F*(1,31) = 3.20	*P* = .09	-1.86	-1.61	.02
	**CP**	***F*(1,31) = 40.88**	***P*<.001**	**2.48**	**1.96**	**.05**
	FM	*F*(1,31) = 3.32	*P* = .08	-1.41	-1.11	.10
	**M**	***F*(1,31) = 12.35**	***P*<.01**	**3.12**	**2.64**	**.30**
	T	*F*(1,31) = 2.31	*P*<.07	-2.16	-1.90	.02
LPP	AF	*F*(1,31) = 4.044	*P* = .05	-1.50	-1.09	.04
	**DF**	***F*(1,31) = 5.76**	***P*<.05**	**-.40**	**-.07**	**.05**
	**CP**	***F*(1,31) = 4.95**	***P*<.05**	**1.38**	**1.13**	**.02**
	**FM**	***F*(1,31) = 5.64**	***P*<.05**	**-.32**	**.063**	**.20**
	**M**	***F*(1,31) = 6.48**	***P*<.05**	**1.42**	**1.06**	**.20**
	O	*F*(1,31) = 3.037	*P*<.09	-.14	-.39	.01

Significant results are in boldface type.

#### Regression of EQ subscales on event-related potentials

The linear prediction of ERP incongruence effects (i.e. incongruent minus congruent potentials) per EQ sub-scale are reported in Table 2.

The incongruence effect in the N400 time window was significantly predicted by the score at the cognitive empathy scale in the left O ROI. The predicted effect reflected a more negative-going curve for participants with high cognitive empathy scores and tended to reverse for lower cognitive empathy scores (cf. Fig 5B). A reversed significant prediction was observable in the left DF ROI, with a less negative-going curve for participants with high cognitive empathy scores. Finally, social skills scores were significantly linked to the N400 effect in the left O ROI, in that they barely tended to disappear or reversed as participants’ social skills scores rise.

## Discussion

This study explored the neuroelectric correlates (ERPs) of pragmatic emotional incongruence (i.e. emotional expectancy violation) effects in healthy participants with various self-reported empathic dispositions. We presented faces displaying basic emotions (or neutral expressions) that were congruent or incongruent with a prior emotional sentential context. The participants were required to judge sentence-facial expression congruence. Our results indicate that pragmatic emotional incongruence is behaviorally more difficult to process than pragmatic emotional congruence (with a greater tendency to make errors and longer response times). Moreover, response times were predictable by self-reported social skills, and difficulties in detecting incongruence appeared greater in participants with low social skills. P100 and N170 components were evoked by facial expressions but, as we expected, incongruence did not modulate these early waves. In this way, our results resemble those of Krombholz et al. [18] who used a paradigm similar to the sentence-picture verification task. The N400 and the LPP waves were affected by pragmatic emotional incongruence, mainly at centro-parietal and centro-posterior midline electrodes sites. In the N400 time window, the amplitude in the left occipital region was linked to the cognitive empathy score, with a more negative amplitude in response to incongruent stimuli among high cognitive empathizers and a reversed pattern for low cognitive empathizers.

### Behavioral effects of pragmatic emotional incongruence

It has been reported that recognizing contextually incongruent facial emotions is more difficult than congruent ones, resulting in more errors [16] and increased response times [10], [16]. In this study, we extended this finding to a situation where the participants judged emotional congruence between a face and a previous sentence describing someone in a situational context.

To our knowledge, we provide the first evidence of incongruence effects on response times linked to self-reported social skills (as defined by Lawrence et al. [23]), in that they would be observable mainly among subjects with lower social skills. Consequently, social skills appeared to be a good predictor of the difficulty participants experienced in detecting pragmatic emotional incongruence with respect to congruence. According to Lawrence et al. [23], “social skills” refer to spontaneity and intuitivism in the process of social information, a construct that was operationalized through Empathy Quotient items such as “I often find it difficult to judge if someone is rude or polite” or “I find it hard to know what to do in a social situation”. Finally, the fact that the ability to detect pragmatic emotional incongruence quickly was predicted by self-reported social skills confirms its importance in everyday social relationships.

### Modulation the N400 by pragmatic emotional incongruence and the influence of empathy

Incongruence is classically found to increase negativity of the N400 wave (supposedly reflecting semantic integration) in centro-parietal regions (for a review, see [24]). In this study, pragmatic emotional incongruence elicited this N400 incongruence effect, not only in the centro-parietal region (with no lateralization effect), but also in the centro-posterior midline region. Thus, we globally replicated the well-established modulation of the N400 concomitant with affective expectancy violations using reading tasks [8], [13]-[14], with especially negative-shifted N400 amplitudes in the case of social expectancy violation or incongruence. In this way, our results confirm that violations of expectations from emotional pragmatic situation models can elicit classic N400 incongruence effects. Our findings as regards the N400 incongruence effect also fit well with the results of a study [18] that employed a paradigm similar to a sentence-verification task. By contrast, studies assessing face priming with affective judgment tasks [10], [16] did not report any classical N400 incongruence effect. To conciliate these findings, we may hypothesize that paradigms requiring participants to make an explicit, conscious judgment on the congruence between the face and the context would facilitate the emergence of classical N400 effects.

The N400 effect found in the antero-frontal ROI, with a less negative N400 for incongruent rather than congruent faces might, in fact, be an electrical counterpart of the N400 incongruence effect found in the CP and M ROIs. In effect, as the average electrical amplitude of all electrodes has been taken as electrical reference, the signal had to be null when averaging all electrodes. Consequently, all incongruence effects had a reversed counterpart.

As we defined cognitive empathy as a way of inferring emotional states, we predicted that the N400 incongruence effect would at least be modulated by self-reported cognitive empathy, similar to Rak et al. [21]. We showed that, in the N400 time window, the amplitude in the left occipital region was predicted by the participants’ scores on the cognitive empathy subscale. Participants with high self-reported cognitive empathy showed more negative amplitudes in the incongruent condition than in the congruent condition. By contrast, regression analyses predicted a reduced or reversed incongruence effect for participants with low self-reported cognitive empathy (less negative-going amplitude in the incongruent condition than in the congruent one). While these modulations in amplitude caused by incongruence might resemble classic and reversed N400 effects, the occipital region is not a brain area where N400 effects are classically described. Consequently, this result requires explanation and replication. Future research exploring the neural sources of this effect with the help of magnetoencephalography or high density EEG might provide some relevant insights.

Besides this link between the cognitive empathy score and the N400 amplitude in the left occipital ROI, N400 amplitude was also linked to the cognitive empathy score in the left dorso-frontal ROI. The pattern appears different in the dorso-frontal region, with an apparently reversed N400 effect in participants with a high cognitive empathy score while participants with low cognitive empathy scores presented null or small classical N400 incongruence effects. However, again, this might be the electrical counterpart of the effect observed in the occipital region. Overall, our results indicate that participants with high cognitive empathy dispositions would process pragmatic emotional incongruence quite differently from those with low cognitive empathy dispositions. Accordingly, N400 incongruence effects are stronger for participants presenting high cognitive empathy scores. In this sense, we replicated the results of studies evidencing that empathy constructs share some of the inter-individual differences with contextual integration of social stimuli [20]-[21]-. Inter-individual differences in solving our experimental task could be related to the fact that some people preferentially simulate others’ emotions either by adopting their perspective (i.e. with the help of cognitive empathy) or by keeping their own perspective. A second way of simulating others’ emotions could be to use general knowledge about how people should feel in a particular situation. We do not exclude that these two ways might co-occur.

The N400 incongruence effect recorded in the left occipital region was also modulated by social skills. The prediction was in favor of a more pronounced N400 incongruence effect in participants with low social skills scores. This appeared contradictory with increased difficulty in processing the task for participants with low social skills scores (evidenced by response time results). One possible explanation is that this result would be an artifact caused by the use of multiple regressions. Indeed, when testing the effect of one variable in the model, all the others predictors in the model remain constant, which modifies data to some extent.

### Modulation of the LPP by pragmatic emotional incongruence

The incongruence effect on the frontal LPP (DF, FM and, marginally, AF ROIs) appeared to be a classic one, with an enhanced amplitude in the incongruence case. Indeed, several late positivities have been reported as being enhanced by incongruence. Along these lines, the LPP is sensitive to a preceding context [29] and has been described as being enhanced by emotional incongruence in affective judgment tasks [10], [16]. The LPP wave has also been found to be enhanced by trait-based expectancy violations in reading tasks [30]-[31]. However, none of the studies listed above as reporting effects on the LPP [10], [16], [30]-[31] used verification tasks. The LPC (Late Positive Complex) is similar to the LPP and has been described as being enhanced by incongruence [32] and related to reanalysis of incongruent stimuli [29], which might be the case in our task. At central and posterior sites, the incongruence effect on the late wave would appear reversed if we consider it as positivity. Like the N400 in the AF ROI, this reversed incongruence effect might be a counterpart, this time of the LPP incongruence effect observed in frontal ROIs. Interestingly, while studies using quite complex reading tasks ([8], [13], [15]) also introduced a character in a sentential way, corresponding to what we did, only one [8] reported a pattern of results similar to ours (incongruence effects on both the N400 and the LPP). It is possible that this would reflect the fact that the situation model generated in that study [8] is more similar to the one generated by our task than the one generated in the other two studies [13], [15].

In the present study, the LPP might also reflect resolution (i.e. understanding of the incongruence) of a previously detected incongruence. This resolution would correspond to an update of the situation model or to the assimilation of the stimulus to the situation model [1]. Nevertheless, this hypothesis is highly speculative because our task did not require resolution of the incongruence between stimuli.

As the emotional reactivity score did not predict any result, the emotional reactivity component appears unrelated to the process of pragmatic emotional incongruence. It is worth noting that the experimental setup was not designed to provide evidence that the task elicited emotional reactivity. The absence of a link between the ERP amplitude and the score with emotional reactivity suggests that such reactivity would not be an automatic process.

## Conclusion

This study evidenced that detecting an incongruent facial expression in a given mentally simulated situational context is more cognitively demanding than detecting a congruent one. It is more difficult to detect pragmatic emotional incongruence than congruence, especially for people with low social skills. This link with self-reported social skills emphasizes the social importance of the ability to detect pragmatic emotional incongruence. While pragmatic emotional incongruence impacted some ERPs (mainly N400 and LPP), we evidenced ERP modulation by one EQ personality dimension, namely cognitive empathy. Further investigation should be conducted in order to better disentangle the neuroelectric correlates of the detection versus the resolution of pragmatic emotional incongruence.

## Supporting Information

S1 Sentences Sentences presented to participants (by emotional category). (DOC)

S1 Supporting Information Significant main hemisphere effects. (DOC)
