# Supplementary material for: Neuroelectric Correlates of Pragmatic Emotional Incongruence Processing: Empathy Matters
Source: PLoS One. 2015 Jun 11;10(6):e0129770. doi: 10.1371/journal.pone.0129770 (PMC4465748; doi:10.1371/journal.pone.0129770)
Supplement: S1 Sentences — (DOC) [file pone.0129770.s001.doc]

**Sentences presented to participants (by emotional category).**

**Joy**

Elle/Il est à la maternité, son bébé vient juste de naître.

She/He is at maternity hospital, her/his baby has just been born.

Elle/Il a gagné quatre médailles d'or aux jeux olympiques.

She/He won four gold medals at Olympic Games.

Elle/Il a rencontré quelqu'un et découvre enfin l’amour.

She/He met someone and finally discovers love.

Elle//Il est parti(e) aujourd'hui pour le voyage de ses rêves.

Today she/he left on the trip of her/his dreams.

Elle/Il a pu gagner le voyage de ses rêves en jouant à un jeu.

She/He was able to win the trip of her/his dreams by participating in a game.

Elle/Il est classé(e) premièr(e) à un concours très difficile.

She/He is ranked the first one in a very difficult competition.

On lui écrit pour lui annoncer une très bonne nouvelle.

We are writing to her/him to announce very good news.

Il fait beau et elle/il a vraiment passé une super journée.

The weather is nice and she/he really had a great day.

Elle/il gagne une très importante somme d’argent au loto.

She/He wins a very big amount of money in the lottery.

Son plus grand rêve vient de se réaliser aujourd'hui.

Her/His greatest dream has just come true today.

Elle/Il prend pour la première fois son enfant dans les bras.

She/He holds for the first time her/his child in her/his arms.

Elle/Il s'est marié(e) hier et part en voyage de noces ce matin.

She/He got married yesterday and is leaving for her/his honeymoon this morning.

Elle/Il a le sentiment d'avoir complètement réussi sa vie.

She/He feels like she/he has been a total success.

Elle/Il était au chômage et vient juste de décrocher un CDI.

She/He was unemployed and has just got a stable job.

Elle/Il a obtenu un financement pour un projet professionnel.

She/He obtained a fund for a professional project.

Elle/Il réalise un projet professionnel qui lui tient à cœur.

She/He realizes a professional project which means a lot to her (him).

Son enfant a enfin obtenu des notes excellentes à l'école.

Her/His child finally got excellent grades at school.

Tout le monde lui fait des compliments ces derniers temps.

Everyone is complementing her/him lately.

Elle/Il apprécie vraiment ses vacances familiales au soleil.

She/He really appreciates her/his family holidays in the sun.

Elle/Il assiste à un bon spectacle comique dans un théâtre.

She/He attends a good comedy show in a theater.

**Fear**

Elle/Il se réveille en sursaut parce que sa chambre est en feu.

She/He wakes up suddenly because her/his bedroom is on fire.

Elle/Il est bloquée dans un bateau à la coque transpercée.

She/He is blocked in boat with a transpierced hull.

La foudre tombe juste à côté d'elle/de lui avec grand fracas.

Lightning falls just next to her/him with big crash.

On la/le menace de mort en braquant une arme à feu sur elle/lui.

Someone is threatening her/him with a gun pointed at her/him.

Elle/il se trouve piégé(e) par une catastrophe naturelle.

She/He gets trapped in a natural disaster.

Elle/Il est pris(e) en otage par de dangereux terroristes.

She/He is hold hostage by dangerous terrorists.

Une voiture lui fonce dessus quand elle/il attend le bus.

A car rushes on her/him while she/he is waiting for the bus.

Une grosse voiture la/le poursuit sur une petite route la nuit.

A big car pursues her/him at night on a small road.

Elle/il est dans un tunnel, un incendie se déclare devant elle.

She/He is in a tunnel, a fire starts in front of her/him.

Elle/Il traverse la rue quand une grosse moto lui fonce dessus.

She/He is crossing a street when a big motorcycle rushes on her/him.

En pleine nuit des inconnus l’interpellent dans une rue.

Strangers call her/him in the middle of the night, out in a street.

La foudre tombe sur le toit de sa maison avec grand fracas.

The lightening falls on her/his house's roof with big crash.

Un chien enragé lui fonce dessus la gueule grande ouverte.

A rabid dog rushes on her/him with its mouth wide open.

Elle/Il est au bord d’un précipice, soudain elle/Il dérape.

She/He is at the edge of an abyss, she/he suddenly slips.

Une épidémie grave se développe rapidement dans sa région.

A serious epidemic quickly progresses in her/his region.

Elle/Il se retrouve seul(e) face à une de ses pires phobies.

She/He ends up alone in front one of her/his worst phobias.

Des voleurs en train de cambrioler sa maison la/le réveillent.

Thieves stealing her/his house wake she/he up.

Elle/Il est suivi(e) dans un souterrain très sombre et désert.

She/He is being followed in a very dark and deserted underground alley.

Elle/Il marche dans un champ de mines antipersonnelles.

She/He is walking in a field of antipersonnel mines.

Elle/Il est dans une forêt, un incendie se déclare à deux pas.

She/He is in a forest, a fire starts next to her/him.

**Sadness**

Elle/Il apprend le décès de quelqu’un qu'elle/Il appréciait.

She/He learns the death of someone she/he appreciated.

Elle/Il doit faire euthanasier son chat qui souffre beaucoup.

She/He has to make euthanize her/his cat that is suffering a lot.

Leur séparation fut très précipitée et elle/il la regrette.

Their separation was very hasty and she/he regrets it.

Des proches viennent de disparaître dans une catastrophe.

Close relations have just disappeared in a disaster.

Un proche lui annonce être atteint d’une maladie mortelle.

A close relation announces to her/him that she/he is suffering from a fatal disease.

Elle/Il vient tout juste de perdre l’amour de quelqu’un.

She/He has just lost someone's love.

Il pleut fort, elle/il apprend le décès subit d'un proche.

It is heavily raining, she/he learns the sudden death of a close relation.

Elle/Il assiste à l'enterrement de quelqu'un de proche.

She/He attends the funerals of a close relation.

Elle/Il est contrainte de vendre la maison de son enfance.

She/He is forced to sell her/his childhood's house.

Elle/Il a l’impression que vraiment personne ne l’aime.

She/He has the impression that really nobody loves her/him.

Elle/Il vient de se rendre compte qu'elle/il a raté toute sa vie.

She/He has just realized she/he totally missed her/his life.

Un proche est très malade, son état empire de jour en jour.

A close relation is very ill, her/his state is worsening from day to day.

Elle/il est devenue paralysée après une rupture d'anévrisme.

She/He became paralyzed after a ruptured aneurysm.

Elle/Il se sent si désemparé(e) par cette atroce nouvelle.

She/He feels so distraught by this atrocious piece of news.

Elle/Il vient juste de perdre l’amitié de quelqu’un de proche.

She/He has just lost the friendship of a close person.

Elle/Il pense que seul(e), jamais plus rien ne sera comme avant.

She/He thinks that alone, nothing will ever be as before.

Elle/Il voit pleurer quelqu'un avec qui elle/il est proche.

She/He sees somebody with whom she/he is close crying.

Elle/Il ne supporte plus d’être tout le temps toute seule.

She/He cannot bear anymore the fact of always being alone.

Quelqu'un qui lui est très proche est en train de déprimer.

A close relationship to him is feeling depressed.

Son chien est mort hier soir alors qu'elle/il était absente.

Her/His dog died yesterday evening while she/he was out.

**Anger**

On vient juste de l’insulter sans aucune raison valable.

Someone has just offended her/him without any valid reason.

Elle/Il risque de payer une amende à la place de quelqu'un.

She/He risks having to pay a fine at the place of somebody else.

Elle/Il est victime d'une agression, personne ne réagit.

She/He is a victim of an aggression, nobody reacts.

On lui reproche quelque chose qu’elle/il n’a jamais fait.

Someone is blaming her/him for something she/he has never done.

Elle/Il apprend qu'on s'est servi d'elle pour une magouille.

She/He learns that somebody used her/him for a scam.

Elle/Il ne supporte plus d'entendre sans cesse des gens crier.

She/He cannot bear anymore hearing people shouting continuously.

Ses vacances ont été reportées pour des raisons injustes.

Her/His holidays have been postponed for unfair reasons.

Elle s'est refait casser le pare-brise de sa voiture.

Someone has broken again her/his car's windscreen.

Elle/Il ne supporte pas du tout qu’on soit injuste avec elle.

She/He cannot stand at all someone being unfair to her/him.

Elle/Il a vraiment fait la queue trop longtemps, inutilement.

She/He was doing the queue for a really long time, pointlessly.

On lui a pris sa place dans une file d'attente interminable.

Somebody took her/his place in an endless waiting queue.

Ca l’énerve qu’on lui dise qu'elle/il a tort, sans explication.

That irritates her/him that people say that she/he is wrong, without explanation.

Elle/Il se dispute violemment avec quelqu’un et ça l’excède.

She/He is having an argument with somebody and that exceeds her/him.

Elle/Il est agacé(e) car il y en a qui la prennent pour un(e) imbécil(e).

She/He is annoyed because some people are taking her/him for an imbecile.

Elle/Il s’indigne que quelqu'un d’élu ait triché à cette fin.

She/He is indignant that one of elected representative cheated to this end.

Elle/Il est complètement excédée, elle va déposer une plainte.

She/He is totally exceeded, she/he will deposit a complaint.

Sa façon de se comporter avec elle/lui l’énerve franchement.

The way she/he behaves with her/him frankly irritates her/him.

Ca l’exaspère qu’on la/le harcèle avec son passé sentimental.

It irritates her/him that people harass her/him with her/his sentimental past life.

Ca l’agace de manquer un rendez-vous pour un retard de train.

It annoys her/him to miss an appointment because of a train delay.

Elle/Il reçoit agacé(e) une amende pour stationnement non payé.

She/He is annoyed to get a fine for an unpaid parking place.

**Neutral**

Elle/Il appuie sur un petit bouton pour ouvrir une porte.

She/He presses on a small button to open a door.

Elle/Il pense qu'elle/Il a pris rendez-vous chez le docteur.

She/He thinks that she/he booked an appointment with the doctor's.

Elle/Il cherche un numéro téléphonique dans les pages jaunes.

She/He looks for a phone number in the yellow pages.

Elle/Il va déposer une lettre dans une boite aux lettres.

She/He is going to deposit a letter in a mailbox.

Elle/Il a conduit une petite voiture de couleur gris clair.

She/He drove a small light gray color car.

Sa tasse à café est dans un petit placard de sa cuisine.

Her/His coffee cup is in a small cupboard in her/his kitchen.

Elle/Il pense qu'un vélo a toujours deux roues et un guidon.

She/He thinks that a bike always has two wheels and a handlebars.

Elle/Il prend toujours son petit-déjeuner dans la cuisine.

She/He always has breakfast in the kitchen.

Elle/Il n'a rien vu de spécial sur la grande route nationale.

She/He saw nothing special on the national main road.

Elle/Il bouge généralement la main droite puis la main gauche.

She/He moves generally the right hand then the left one.

Elle/Il prend une feuille blanche et un stylo bille noir.

She/He takes a white sheet and a black pen ball.

Le soir elle/il éteint la lumière de sa chambre à coucher.

In the evening, she/he switches off the light of her/his bedroom.

Elle/Il penche la tête en avant et regarde en bas de chez elle.

She/He bends the head forward and looks down from his home.

Elle/Il entend les gens discuter entre eux dans la ruelle.

She/He hears people discussing in the alley.

Elle/Il désire un nouveau réveil pour mettre dans sa chambre.

She/He wishes for a new alarm clock to put in her/his bedroom.

Elle/Il est sur le chemin de sa réunion du lundi après-midi.

She/He is on the way to her/his Monday afternoon's meeting.

Elle/Il verra bien ce qu'elle fera chez elle/il demain soir.

She/He will see well what he will do home tomorrow evening.

Elle/Il est assise dans sa chambre, devant son ordinateur.

She/He sits in her/his bedroom, in front of her/his computer.

Elle/Il regarde la table basse noire à côté de son bureau noir.

She/He looks at the black coffee table next to her/his black desk.

Elle/Il est debout dans son salon et regarde dans le vague.

She/He stands in her/his living-room and looks in the vagueness.
