# Supplementary material for: Neuroelectric Correlates of Pragmatic Emotional Incongruence Processing: Empathy Matters
Source: PLoS One. 2015 Jun 11;10(6):e0129770. doi: 10.1371/journal.pone.0129770 (PMC4465748; doi:10.1371/journal.pone.0129770)
Supplement: S1 Supporting Information — (DOC) [file pone.0129770.s002.doc]

**Significant main hemisphere effects.**

| Wave | ROI | F value | Significance | Amplitude (µV) | η² |
| --- | --- | --- | --- | --- | --- |
| P100 | CP | *F*(1,31)=5.14 | *P*<.05 | L=-0.14  R=0.21 | .1 |
|  | T | *F*(1,31)=4.99 | *P*<.05 | L=.060  R=.43 | .07 |
| N170 | O | *F*(1,31)=4.49 | *P*<.05 | L=-.19  R=-1.04 | .1 |
| P300 | CP | *F*(1,31)=10.80 | *P*<.01 | L=1.05  R=1.77 | .2 |
| N400 | CP | *F*(1,31)=16.33 | *P*<.001 | L=1.59  R=2.84 | .3 |
|  | DF | *F*(1,31)=14.19 | *P*<.001 | L=-2.10  R=-1.36 | .2 |
| Late wave | O | *F*(1,31)=11.67 | *P<.01* | L=.219  R=-.754 | .2 |
|  | T | *F*(1,31)=7.07 | *P<.05* | L=-.389  R=-1.21 | .1 |
